# Liquid-Metal Core–Shell Particles Coated with Folate and Phospholipids for Targeted Drug Delivery and Photothermal Treatment of Cancer Cells

**DOI:** 10.3390/nano13132017

**Published:** 2023-07-06

**Authors:** Suyeon Ahn, Seung Hyun Kang, Hyunjeong Woo, Kyobum Kim, Hyung-Jun Koo, Hee-Young Lee, Yonghyun Choi, Shin Hyuk Kang, Jonghoon Choi

**Affiliations:** 1 School of Integrative Engineering, Chung-Ang University, Seoul 06974, Republic of Korea; awert9717@gmail.com (S.A.); hyunjeong1226@gmail.com (H.W.); 2 Departments of Plastic and Reconstructive Surgery, Chung-Ang University Hospital, Chung-Ang University College of Medicine, Seoul 06973, Republic of Korea; kangsh0708@cauhs.or.kr; 3 Department of Chemical & Biochemical Engineering, Dongguk University, Seoul 04620, Republic of Korea; kyobum.kim@dongguk.edu; 4 Department of Chemical and Biomolecular Engineering, Seoul National University of Science and Technology, Seoul 01811, Republic of Korea; hjkoo@seoultech.ac.kr; 5 Department of Chemical Engineering, Kumoh National Institute of Technology, Gumi-si 39177, Republic of Korea; lhysshr@kumoh.ac.kr; 6 Feynman Institute of Technology, Nanomedicine Corporation, Seoul 06974, Republic of Korea

**Keywords:** liquid metal, eutectic gallium indium, cancer targeting, folate, photothermal, drug delivery system

## Abstract

Recently, several methods have been used for cancer treatment. Among them, chemotherapy is generally used, but general anticancer drugs may affect normal cells and tissues, causing various side effects. To reduce the side effects and increase the efficacy of anticancer drugs, a folate-based liquid-metal drug nanodelivery system was used to target the folate receptor, which is highly expressed in cancer cells. A phospholipid-based surface coating was formed on the surface of liquid-metal nanoparticles to increase their stability, and doxorubicin was loaded as a drug delivery system. Folate on the lipid shell surface increased the efficiency of targeting cancer cells. The photothermal properties of liquid metal were confirmed by near-infrared (NIR) laser irradiation. After treating cancerous and normal cells with liquid-metal particles and NIR irradiation, the particles were specifically bound to cancer cells for drug uptake, confirming photothermal therapy as a drug delivery system that is expected to induce cancer cell death through comprehensive effects such as vascular embolization in addition to targeting cancer cells.

## 1. Introduction

Many people around the world die from cancer, which is the leading cause of death in the world [1]. Cancer is characterized by self-sufficiency in growth signals and insensitivity to anti-growth signals. It has limitless replicative potential, with no finite lifespan, and the ability to evade apoptosis. It is also characterized by sustained angiogenesis, which allows it to invade and metastasize to other tissues [2]. Chemotherapy is generally used for the treatment of these cancers. However, if a general anti-cancer drug is prescribed instead of a targeted anti-cancer drug, it circulates throughout the body, affecting not only cancer cells but also normal cells, causing significant side effects such as vomiting, hair loss, fatigue, and cognitive decline [3,4]. Therefore, an attempt was made to prepare particles capable of controlling the release action of anticancer drugs by external stimuli [5]. Recently, many studies related to the production of folate-conjugated nanoparticles to target cancer cells have been conducted [6,7]. We aimed to induce cancer cell death by increasing the delivery of existing anticancer drugs by targeting the folate receptor, which is present in most cancer cells and relatively overexpressed in breast cancer, in order to reduce the side effects of existing anticancer drugs.

**Figure 1:**
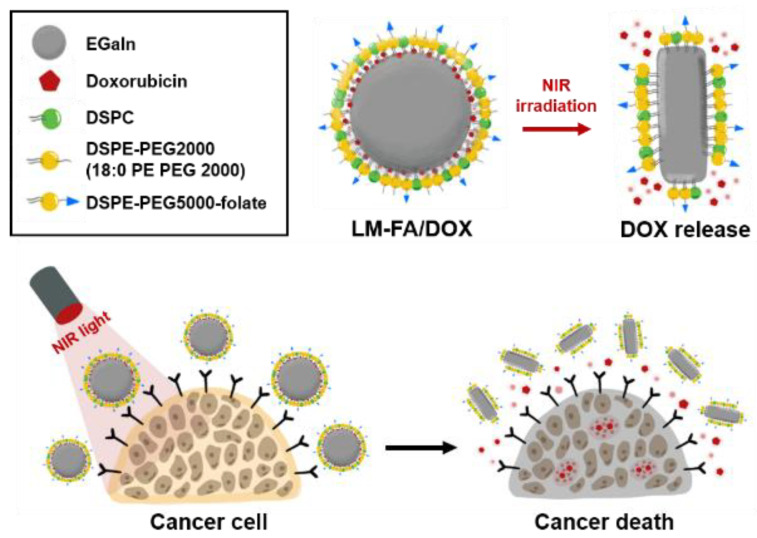
Experimental scheme based on particle shape change and controlled drug release by NIR irradiation.

To utilize nanoparticles with biocompatible properties, we used eutectic gallium indium (EGaIn), a liquid metal. Mercury, a representative liquid metal, is toxic and difficult to use in living organisms, so EGaIn, an alloy of gallium and indium in a certain proportion, has become a focus of recent studies [8]. Liquid metals, which are liquid at room temperature, are mostly characterized by their fluidity and conductivity. EGaIn is widely used in electrical circuits and sensors and as a contrast agent in computed tomography and X-ray [9,10]. It also has a photothermal effect, giving off heat under near-infrared (NIR) irradiation [11]. Photothermal therapy is a non-invasive treatment method that kills cancer cells by converting light energy into heat energy [12,13]. EGaIn easily becomes particulate via sonication, and the size of the particles can be adjusted according to the processing time [11,14,15]. We modified the surface phospholipid for increased stability and decreased toxicity of the EGaIn nanoparticles and loaded doxorubicin, a drug for treating malignant tumors, for drug delivery (Figure 1). Surface modification of folate to target the folate receptor expressed in cancer cells can improve drug delivery efficiency due to intracellular signaling of the folate receptor, which leads to an increase in the cancer suppressor protein P53, thus enabling cancer cells to die more quickly [16]. In addition, when irradiated under NIR, the shape is changed by the photothermal feature of the liquid metal, and when the drug is released, cellular activity in the area is activated, which can speed up drug uptake. Through this synergistic anti-cancer effect, we wanted to induce rapid death of cancer cells and help treat cancer patients in the absence of specialized drugs.

## 2. Materials and Methods

### 2.1. Materials

All reagents, unless otherwise specified, were purchased from Sigma-Aldrich (St. Louis, MO, USA). Reagents 18:0 PC, 1,2-distearoyl-sn-glycero-3-phosphocholine (DSPC), 18:0 PEG2000 PE, and 1,2-distearoyl-sn-glycero-3-phosphoethanolamine-N- [methoxy(polyethylene glycol)-2000] (DSPE-PEG2000) were obtained from Avanti Polar Lipid, Inc. (Alabaster, AL, USA). Reagent 1,2-distearoyl-sn-glycero-3-phosphoethanolamine-N- [folate(polyethylene glycol)-5000] (DSPE-PEG5000-Folate) was purchased from Nanosoft Polymers (Winston–Salem, NC, USA). HeLa and human dermal fibroblast (HDF) cells were obtained and cultured from Korea Cell Link Bank (Seoul, Korea). MDA-MB-231 breast cancer cells were purchased and cultured from the American Type Culture Collection. Dulbecco’s modified Eagle medium (DMEM, high glucose) was purchased from Pan Biotech (Aiden Bach, Germany). DMEM (low glucose), Dulbecco’s phosphate-buffered saline (DPBS), fetal bovine serum (FBS), and antibiotic-antimycotic (100×) were purchased from Gibco (Carlsbad, CA, USA). Calcein AM, EthD-1, Hoechst 33342 (trihydrochloride), Vybrant™ DiO cell-labeling solution, and Human p53 ELISA Kit were purchased from Invitrogen (Thermo Fisher Scientific Inc., Waltham, MA, USA). Pierce™ RIPA Buffer was purchased from Thermo Fisher Scientific. Human FOLR1 Alexa Flour 488-Conjugated Antibody was purchased from R&D Systems, Inc. (Minneapolis, MN, USA).

### 2.2. Synthesis of Liquid Metal Nanoparticles

To obtain LM/DSPC/DSPE-FA/DOX (hereafter LM-FA/DOX) particles, a 50 mL disposable glass vial containing 10 mg EGaIn, 750 µL DSPC (25 mg/mL, in chloroform), 18:0 PEG2000 PE 600 µL (25 mg/mL, in chloroform), DSPE-PEG (5000) Folate 500 µL (2 mg/mL, in chloroform), and DOX 1 mg was added and dried in a 70 °C oven for 2 h to volatilize the chloroform. For EGaIn particles without phospholipids, 10 mg EGaIn was added to a 50 mL disposable glass vial. Then, 10 mL of deionized (DI) water was added and dispersed for 10 min at 60 °C bath sonication above the transition temperature (55 °C). Because EGaIn is sensitive to heat, the vial containing EGaIn was placed on ice and dispersed using a tip sonicator for 1 h to form particles. This was followed by a washing process consisting of centrifugation at 12,000× *g*, 4 °C for 10 min to remove phospholipids that were not involved in particle formation. The supernatant was removed and dispersed with DI water to the required particle concentration.

### 2.3. Characterization

The size and concentration of each particle were determined using nanoparticle tracking analysis (NTA) (Nanosight LM10 NTA, Malvern, UK). All samples were measured in appropriate dilutions, and particle counts were calculated by reflecting the dilution factor in the final concentration. In addition, the size and zeta potential of each particle were determined using dynamic light scattering (DLS) (Zetasizer Pro, Malvern, UK). The laser type is He-Ne (633 nm), and the scatter detector angle for size measurement is 173°. All samples were diluted 20-fold and placed in a dedicated Zetasizer cell for measurement. The measurements were performed in triplicate with a refractive index of 3.9 and an absorption of 0.13. To confirm the size and morphology of the synthesized particles, field emission transmission electron microscope (FE-TEM) analysis was performed (EM-F200(TFEG); JEOL Ltd., Tokyo, Japan). For analysis, the samples were placed on a copper grid, dried, and analyzed at a high voltage of 200 kV. Staining was not performed, as there is a clear difference in brightness between metal and phospholipid membranes. FT-IR analysis was performed to analyze the functional groups on the surface using an Alpha II spectrometer (Bruker, Billerica, MA, USA). The baseline was measured with a blank surface without placing the sample on the measurement part. To confirm the optical properties of the particles, a UV/Vis spectrophotometer (Nabi, MicroDigital Co., Ltd., Seongnam-si, Republic of Korea) spectrum mode was used. The samples were measured in a cuvette (UV/Visible range Cuvettes) at 350 nm and 900 nm. DI water was used as the blank.

### 2.4. DOX-Loading Efficiency and In Vitro Drug Release Test

For quantitative analysis of doxorubicin (DOX) loaded on the particles, a standard curve was obtained at 480 nm wavelength by serial dilution of 1 mg/mL stock solution (in DI water). To obtain the loading efficiency of the drug during the synthesis of LM-FA/DOX particles, the supernatant of free DOX that could not be loaded into the sample was separated by centrifugation at 12,000× *g*, 10 min, 4 °C. This was serially diluted to the same degree as the standard curve, and the absorbance of free DOX was measured using a multi-plate reader (Synergy H1, BioTek) at 480 nm wavelength. The absorbance of free DOX relative to the absorbance of the standard curve was used to obtain the concentration, and the drug-loading efficiency was calculated by applying the formula below.

$$\mathrm{EE}\left(\%\right)=\frac{\mathrm{Initial}\;\mathrm{mass}\;\mathrm{of}\;\mathrm{drug}-\mathrm{free}\;\mathrm{doxorubicin}\;\mathrm{mass}}{\mathrm{Initial}\;\mathrm{mass}\;\mathrm{of}\;\mathrm{drug}}\times 100$$

In addition, to obtain the concentration of drug released by DOX loaded on LM-FA/DOX particles as a function of incubation time, 1 mL LM-FA/DOX was placed in a dialysis bag, which was then added to 10 mL of 30% ethanol as an external buffer. The drug released from 1 mL was then incubated, and the drug release concentration was measured over time.

### 2.5. NIR Treatment

To confirm the photothermal properties of the synthesized particles, we conducted a contrast experiment using an infrared LED module (SLM-85560). The infrared LED module is 2.4 W, 1 A, and emits a wavelength of 855 nm. The synthesized particles were placed in 200 µL in an e-tube at a concentration of 100 µg/mL and measured at 30 s intervals, and three replicates were performed for each experimental group.

### 2.6. Cell Cytotoxicity Test

Three cell lines were used: HeLa, MDA-MB-231, and HDF. For the cell cytotoxicity test, cells were seeded in 96 wells at a concentration of 1 × 10^5^ cells/mL, 100 µL per well. The medium was high/low-glucose DMEM (10% FBS, 1% penicillin, streptomycin). Cells were cultured in an incubator at 37 °C, 5% CO_2_. After 24 h, 100 μg/mL liquid metal was treated in each sample. Here, 1% Triton X-100 was used as a positive control. After 24 h, the samples were washed once with phosphate-buffered saline (PBS), treated with 10% Cell Counting Kit-8 (CCK-8) reagent, and incubated for 2 h in an incubator at 37 °C, 5% CO_2_. Then, samples were analyzed by measuring the absorbance at 450 nm with a multi-plate reader.

To check the cell cytotoxicity when the particles were at a concentration of 100 µg/mL, we followed the same procedure as above. On day 3, instead of using CCK-8 reagent, Hoechst at 1 µg/mL and calcein AM at 0.5 µg/mL were mixed in PBS to a concentration of 100 µL per well. After incubation at 37 °C, 5% CO_2_ for 20 min, the wells were washed twice with PBS and measured at λEm 485 nm and λEx 520 nm for calcein AM and λEm 360 nm and λEx 460 nm for Hoechst with a multi-plate reader.

Cell cytotoxicity was further confirmed by fluorescence microscopy. Each cell line was seeded in 48 wells at a concentration of 2.5 × 10^4^ cells/mL, 500 µL per well. Otherwise, the procedure was the same as previously described.

### 2.7. FACS Measurement

To determine the expression of folate receptor 1 (FOLR1) on the cell surface, FACS was measured using Alexa-488 conjugated FOLR1 antibody. MDA-MB-231, HeLa, and HDF cells were prepared and suspended in PBS containing 5% FBS at 1 × 10^6^ cells/100 μL. Then, 1 µg of FOLR1 antibody was added and incubated under ice, in dark room conditions. After 50 min, the cells were centrifuged at 3000 RPM for 4 min. The supernatant was removed to isolate the unbound free antibody. The cell pellet was resuspended with 500 µL PBS containing FBS and transferred to a FACS tube to measure binding using FACS (Aria Ⅱ).

### 2.8. P53 ELISA

HeLa, MDA-MB-231, and HDF cell lines were used. Cells were seeded in 12 wells at a concentration of 1 × 10^5^ cells/mL, 1 mL per well. The medium was high/low-glucose DMEM (10% FBS, 1% penicillin, streptomycin). Cells were cultured in an incubator at 37 °C, 5% CO_2_. After 24 h, 100 µg/mL liquid metal was treated in each sample. After 24 h of incubation, the medium was removed and washed with DPBS, and then 500 µL of RIPA buffer was added to each well, wrapped in foil, and placed in a 4 °C refrigerator. After 15 min, the cells were scraped with a cell scraper and centrifuged at 8000× *g*, 4 °C for 20 min. Since P53 is an intracellular protein, the supernatant was used. The enzyme-linked immunosorbent assay (ELISA) plate treated with capture antibody was washed once with wash buffer, and then 100 µL of the sample and 100 µL of biotin-conjugated antibody were added to each well. After incubation at room temperature for 2 h, the plate was washed three times to remove the unbound sample and antibody. Streptavidin-HRP was then added and left to react for 30 min. The absorbance of the sample was checked with TMB solution at 450 nm. Because the absorbance was measured in proportion to the concentration of P53, the difference in the concentration of P53 can be determined.

### 2.9. Cancer Targeting Effect Test

Since the surface of the synthesized particles has a phospholipid membrane, we checked for cell binding after fluorescent staining with DiO, which stains phospholipids. First, 5 µL DiO was added to 1 mL of the synthesized particles to a concentration of 0.005 mM/mL. Then, incubation was carried out at 37 °C, in the dark. After 30 min, the cells were centrifuged at 12,000× *g* for 10 min and at 4 °C and washed three times to ensure that only DiO-stained particles remained. The cells were resuspended in a final volume of 300 µL PBS at a concentration of 2 × 10^5^ cells/mL and LM at a concentration of 300 µg/mL. The cells were then incubated at 37 °C, 5% CO_2_, in the dark with shaking on an orbital shaker for 1 h. After the cells were suspended, they were washed three times by centrifuging at 1000× *g* and 4 ℃ for 3 min. The fluorescence of the cell-bound particles was checked at λEm 485 nm and λEx 520 nm with a multi-plate reader.

### 2.10. Cell Viability Test (Live & Dead Assay, Trypan Blue Assay)

To confirm the effectiveness of drug release using the photothermal properties of the synthesized particles, the particles were illuminated using an infrared LED module (SLM-85560) and then subjected to live and dead assays on the HeLa, MDA-MB-231, and HDF cell lines. Confocal dishes were seeded at a concentration of 1.5 × 10^5^ cells/mL, 2 mL per well. The medium was high/low-glucose DMEM (10% FBS, 1% penicillin, streptomycin). Cells were cultured in an incubator at 37 °C, 5% CO_2_. After 24 h, each cell was treated with a concentration of 25 µg/mL of the synthesized particles (LM-FA/DOX) and irradiated for 10 min using an infrared LED module, with three replicates. After NIR irradiation, the cells in the confocal dish were treated with Hoechst at a concentration of 1 µg/mL and calcein AM at a concentration of 0.5 µg/mL in PBS, 100 µL per well. Fluorescence images were taken using a STELLARIS 5 confocal microscope (LEICA, Munich, Germany).

To quantitatively determine the efficacy of drug release through the photothermal properties of LM-FA/DOX particles, a trypan blue assay was performed. HeLa, MDA-MB-231, and HDF cell lines were used to seed 96 wells at a concentration of 1 × 10^4^ cells/mL, 100 µL per well. The medium was high/low-glucose DMEM (10% FBS, 1% penicillin, streptomycin). Cells were cultured in an incubator at 37 °C, 5% CO_2_. After 24 h, 100 µg/mL liquid metal was treated in each sample. After 2 h, the particles that were not bound to the cell were washed with PBS, and after 22 h, the cells were irradiated with NIR for 1 min, 2 min, 5 min, and 10 min. After treating each well with 50 µL trypsin, the cells were detached and stained with trypan blue. The number and percentage of live and dead cells were then counted using JuLI™ Br bright field cell history recorder (Seoul, Korea).

### 2.11. Statistics

Two-tailed unpaired *t*-tests were conducted in GraphPad Prism 7.0 for Windows (GraphPad Software, Inc., La Jolla, CA, USA). Each experiment was performed in triplicate, with error bars indicating standard deviations. Non-significant values are represented as ns, while *, **, ***, and **** indicate *p*-values < 0.1, 0.01, 0.001, and 0.0001, respectively.

## 3. Results and Discussion

### 3.1. Liquid Metal Particle Characterization

Liquid metal generally refers to metals that are liquid at room temperature. There are many types of liquid metals, but among them, EGaIn has been utilized in various fields due to its properties such as flexibility, low toxicity, biocompatibility, and photothermal effect. In this study, we aimed to increase the stability by forming a surface film using phospholipids on the surface of the particles, using it as a drug delivery system by loading DOX onto the particles.

**Figure 2:**
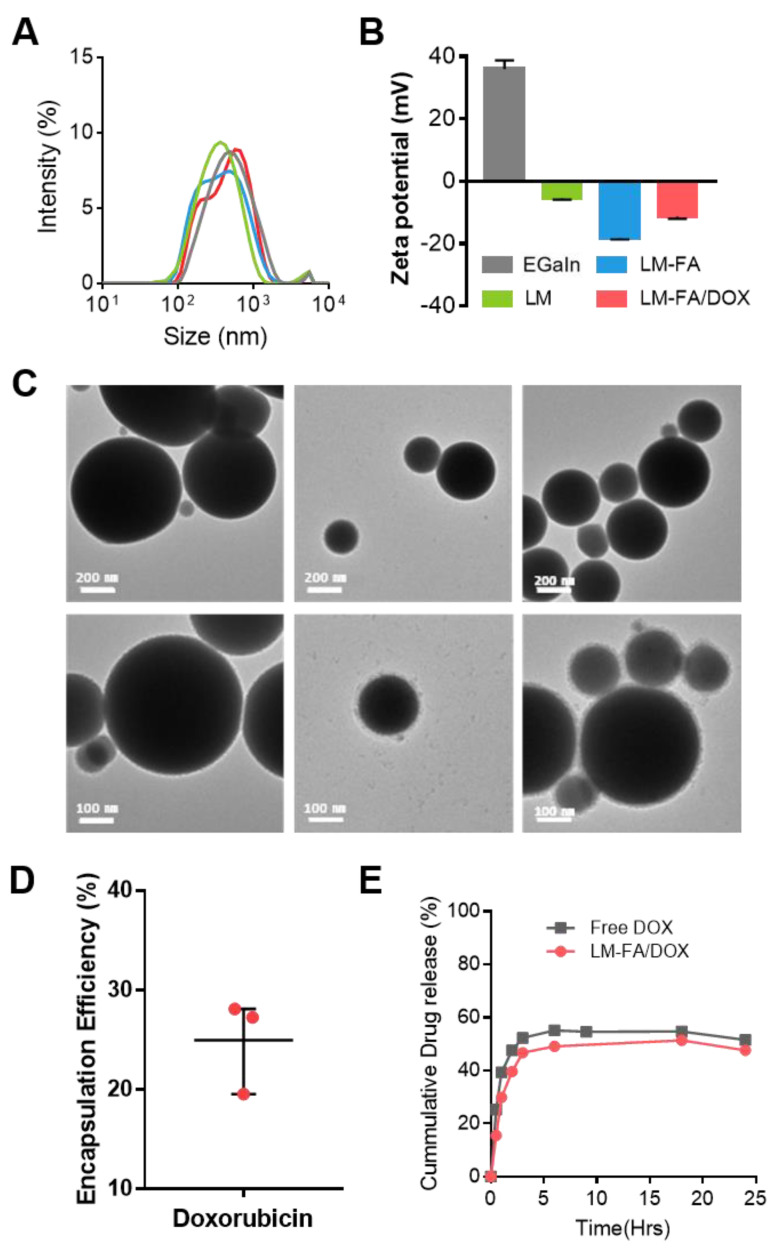
**LM particle characterization.** (**A**) NTA size data. (**B**) DLS Zeta potential data; Gray: EGaIn; Green: LM; Blue: LM-FA; Red: LM-FA/DOX. (**C**) TEM; from left, EGaIn, LM, and LM-FA. (**D**) Encapsulation efficiency of LM-FA/DOX. (**E**) In vitro cumulative doxorubicin release efficiency from LM-FA/DOX at ethanol 30% buffer.

To increase the stability of EGaIn particles, we formed core–shell particles with phospholipids wrapped on the outside. Four types of particles—EGaIn, LM, LM-FA, and LM-FA/DOX—were prepared for comparative analysis. The average particle size of the four types is 300 nm and in DLS distribution. In general, PDI values below 0.3 are acceptable in terms of particle size uniformity [17]. When checking the PDI value of LM-FA particles compared to EGaIn, EGaIn had an average value of 0.304, while LM-FA had a value of 0.269. This confirms that LM-FA can be fabricated into particles with uniform size by phospholipid compared to EGaIn. In addition, the concentration of each particle is, on average, between 1 × 10^9^ and 10^10^ particles/mL when measured by NTA. This indicates that the particles are formed at a constant concentration and size. To determine if a phospholipid film had formed on the surface of the particles, the zeta potentials were compared. EGaIn has a positive charge, but when a phospholipid layer is formed, the value decreases to a negative charge. EGaIn has a positive charge value of +35.96 mV, and when a phospholipid layer is formed, it has a negative charge value. It was confirmed that LM had −5.31 mV, LM-FA had −18.13 mV, and LM-FA/DOX had −11.29 mV. This indicates that it is a core–shell particle with a phospholipid layer on the surface. Furthermore, LM-FA has the strongest negative charge value, indicating that folate is present on the surface, which affects the zeta potential value (Figure 2A). This is because a larger amount of phospholipid is added to LM-FA than LM. It can be inferred that the DSPE-PEG5000-folate phospholipid added to modify the folate results in a lower zeta potential at the interfacial region than LM [18,19]. The core–shell morphology was confirmed by TEM and SEM analysis which showed a liquid metal core surrounded by the phospholipid layer (Figure 2B and Figure S1A). In addition to visually confirming the presence of phospholipids on the surface of the particles, we analyzed the surface modification by identifying functional groups on the surface of the particles through FT-IR. As a result, we found peaks at 1450 cm^−1^, 2922 cm^−1^, and 3025 cm^−1^ in LM-FA compared to EGaIn (Figure S1D). The C-H bending and C-H stretching structures, which represent alkanes, were confirmed by reference, indicating alkanes among the phospholipid structures (DSPC, DSPE-PEG) bound to the surface. These results confirm that the weight ratio of C is about 14% in EDS mapping (Figure S2).

To confirm the optical characterization of EGaIn and LM-FA particles, UV-Vis-NIR absorbance spectra were compared before and after irradiating the particles with NIR. Particles with EGaIn and LM-FA particles with phospholipid and folate surface modification were analyzed from 350 nm to 900 nm. The results showed that the particles had high absorbance at all wavelengths, and the spectral shapes of the particles were similar for EGaIn and LM-FA, confirming that the particles were made of the same material. In addition, the spectral shapes were the same before and after NIR irradiation, indicating that the particles were highly stable. This shows that EGaIn is a particle that can absorb almost all light, and when irradiated with 855 nm NIR, it can absorb light and produce a photothermal effect (Figure S1C).

To kill the cancer cells, liquid metal particles were loaded with DOX at the same time that the phospholipid was wrapped on the outside. The particles were synthesized by adding 1 mg to encapsulate DOX in the particles. To check the loading efficiency of DOX, the free DOX that could not be loaded onto the particles was separated, and its absorbance was measured. The absorbance measurements were used to create a DOX standard curve, and linear regression analysis showed that the loading efficiency was around 25%, on average (Figure 2D).

The amount of drug released from the particles was checked for 24 h by placing the particles in a dialysis bag in a 30% ethanol external buffer. For the control group (Free DOX), we calculated the encapsulation efficiency and prepared a solution with the same DOX concentration. As a result, we found that the amount of DOX released from the particles was approximately 50% of that of the control group (Figure 2E). This demonstrated that DOX drug release from the particles was possible. Due to the refraction of NIR laser light by the external buffer, ethanol 30% was used to indirectly check the possibility of drug release in the particles. To confirm that controlled release is possible, we also checked the drug release in PBS, but no drug was released. This indirectly confirmed that the controlled release of the drug was possible. Furthermore, the decay plot of drug release shown at the end indicates that very little of the drug was released in the 4 h from 20 to 24 h. This suggests that by about hour 20, all of the loaded drug had been released.

### 3.2. Photothermal Characterization by NIR Irradiation

EGaIn is an alloy with 75% gallium and 25% indium by weight, which has low toxicity and good biocompatibility. LM particles using EGaIn can undergo morphological changes due to heat. This is because the heat transitions from Ga to GaOOH present in EGaIn and induce a transformation from spherical to rod morphology [20,21]. This explains the slight increase in O in the composition of LM-FA (Figure S2). LM particles, such as gold nanostructures, have a photothermal effect. This is the property of converting light into heat, and liquid metals can be used as photothermal agents [21].

**Figure 3:**
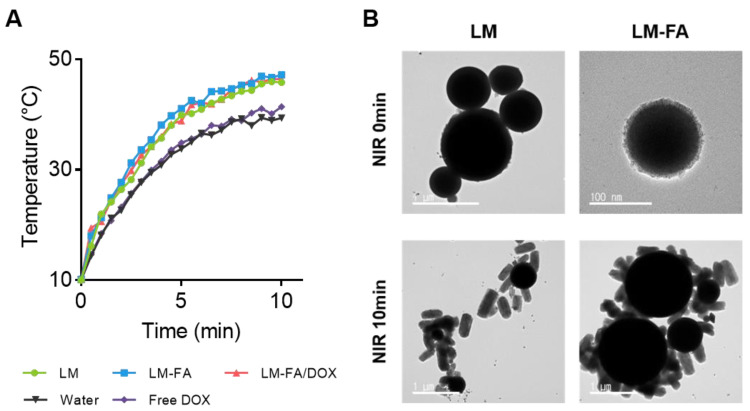
**Temperature and morphology change by NIR irradiation time.** (**A**) Rapid temperature change in LM particles. (**B**) Shape transfer of LM particles.

We previously showed that EGaIn and LM-FA have high absorbance in the absorbance spectrum (Figure S1C). To confirm the photothermal characteristics of LM, LM-FA, and LM-FA/DOX particles, 855 nm NIR was used among the wavelength ranges of lasers that can be irradiated to the human body [22]. In water and free DOX, the temperature increased to about 40 °C, and it took 10 min to reach the maximum temperature. On the other hand, in the presence of particles, the temperature increased to about 48 °C. In addition, the time taken for water to reach its peak temperature was reduced by half, to about 5 min (Figure 3A). Compared to water and free DOX, which are control groups, the temperature increased quickly in the particles containing EGaIn. Through this, it was confirmed that the LM had an ideal photothermal conversion efficiency.

Furthermore, FE-TEM was used to confirm that photothermal properties were present under NIR irradiation. LM and LM-FA particles were fabricated and irradiated with NIR for 5 min, the time it takes to reach the maximum temperature, and then analyzed by FE-TEM. As a result, we found that both LM and LM-FA particles changed their shape from spherical to rod-shaped (Figure 3B), indicating that the photothermal effect of LM particles resulted in a faster, more stable temperature rise and morphology change.

### 3.3. Cytotoxicity and Cancer Cell Targeting Effect by NIR Irradiation Time

Since many metal particles are toxic [23,24,25], a cytotoxicity test was conducted to confirm the toxicity of EGaIn particles. Cells were treated with different concentrations of LM particles to establish the lowest LM concentration that did not affect cells. We performed the CCK-8 assays using three cell lines: HeLa, MDA-MB-231, and HDF. We examined the effects at concentrations of 500, 250, 100, 50, and 10 µg/mL and found that all cell lines retained approximately 90% or more viability (Figure S3). After performing the CCK-8 assay, we checked the condition of the cells under a microscope and found that 100 µg/mL was the concentration with the best morphology and least impact on the cells. All subsequent cell experiments were performed at the 100 µg/mL concentration.

**Figure 4:**
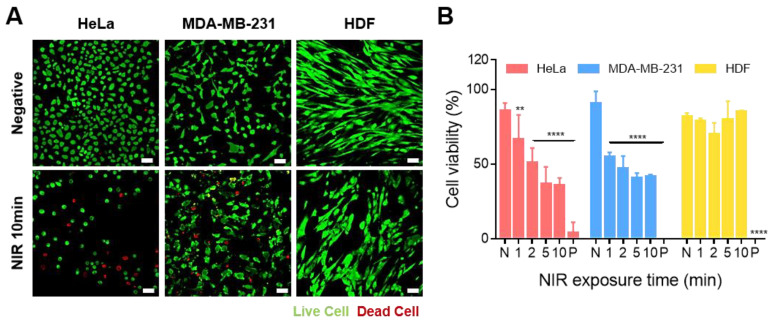
**Effect of LM-FA/DOX on cell viability according to NIR irradiation time.** (**A**) Live and dead assay (HeLa, MDA-MB-231, and HDF). (**B**) Cell viability at 100 µg/mL of particle concentration through trypan blue assay. All statistical significance was confirmed for the negative control. Non-significant values have been represented as ns, while **, and **** indicate *p*-values < 0.0021 and 0.0001, respectively.

We checked the drug release with and without NIR irradiation and found that the particles were specifically bound to cancer cells by folate attached to the particles (Figure 4). Using this specific binding to target cancer cells, we checked the cell viability by drug release according to the NIR irradiation time to confirm the drug deliverability according to the NIR irradiation time. Quantitative and qualitative experiments of live and dead cells were conducted through live and dead assay and trypan blue assay. Live and dead assays were analyzed by confocal microscopy and trypan blue assays were averaged using two methods: direct counting and JuLI Br. HeLa and MDA-MB-231 cells showed a tendency to have more dead cells after 10 min of NIR irradiation. On the other hand, HDF cells did not show many dead cells despite NIR irradiation. This trend was also seen in the trypan blue assay.

As a result, the number of dead cells increased in cancer cells HeLa and MDA-MB-231 depending on the NIR irradiation time. The above trend confirmed that folate on the surface of liquid metal particles can be targeted to cancer cells and that the photothermal effect of liquid metal particles can be used to induce drug release and kill cancer cells.

Furthermore, photothermal treatment of cancer cells is possible using liquid-metal core–shell particles. The liquid-metal particles were modified with folate to target the folate receptor, which is specifically expressed in cancer cells, and DOX was loaded onto the particles to release the drug at the target site. We confirmed the photothermal effect, in which the drug is released as the shape of the particle changes under NIR irradiation. We took advantage of the release of drugs from liposomes due to the photothermal effect using NIR [26]. Therefore, we aimed to fabricate nanoparticles that can effectively inhibit tumor growth by enabling the controlled release of drugs from particles through NIR irradiation [27].

### 3.4. Cellular Uptake of LM-FA/DOX

To evaluate the feasibility of drug delivery, we observed with a confocal microscope whether the drug released from the particles was uptaken by the cells. After calculating the encapsulation efficiency of LM-FA/DOX particles, the same concentration of DOX solution was prepared and the cells were stained after a 2 h uptake. To confirm that the drug release from LM-FA/DOX particles can be controlled with or without NIR irradiation, we checked how much DOX was taken up by HeLa, MDA-MB-231, and HDF cells after 5 min of NIR irradiation.

**Figure 5:**
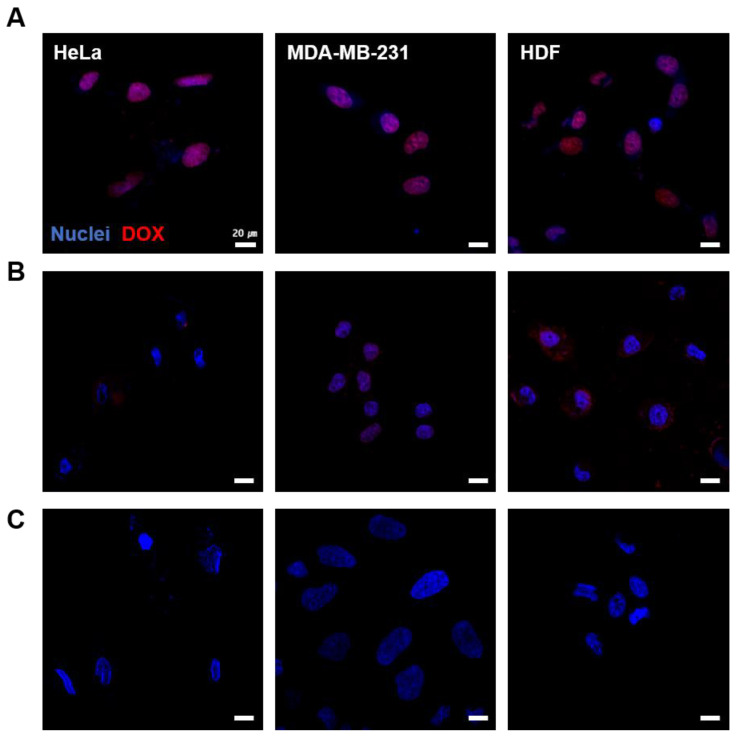
**Cellular uptake test of DOX by NIR irradiation.** From left: HeLa, MDA-MB-231, and HDF cell. (**A**) Free DOX uptake. (**B**) DOX uptake by LM-FA/DOX particles (no irradiation NIR). (**C**) DOX uptake released by NIR irradiation.

Free DOX was confirmed to be taken up into the nuclei of the cells by the fluorescence of the drug (Figure 5A), whereas it was difficult to see drug uptake in the LM-FA/DOX particles that had not been irradiated with NIR (Figure 5B,C). After 5 min of NIR irradiation, the drug was shown to be taken up, suggesting that drug release is controlled by the presence or absence of NIR irradiation. It can be inferred that the shape of LM-FA/DOX particles changes from spherical to rod-shaped upon NIR irradiation due to the photothermal nature of the particles, resulting in the release of the drug. This confirms drug delivery to cancer cells using the photothermal effect. After being absorbed into DNA, DOX induces cell death and causes DNA damage through several mechanisms, including cell cycle arrest, inhibition of DNA repair mechanisms, and generation of free radicals [28]. Confocal microscopy showed that DOX was delivered to the cell nucleus, as the area where the nucleus was stained exactly coincided with the area where DOX was introduced (Figure S4). On the other hand, no drug release occurred from LM-FA particles that were not irradiated with NIR. Recently, a drug delivery system targeting the folate receptor is being studied, and based on the above results [29], we have produced particles that have DOX delivery powder specifically for cancer cells.

### 3.5. In Vitro Cell Expression by Liquid Metal Particle

**Figure 6:**
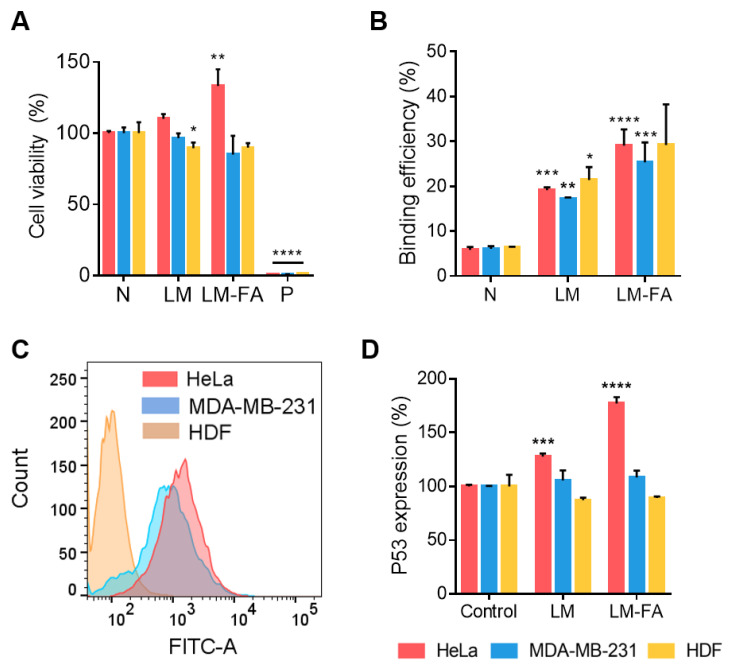
**Effects of LM particles on the cells.** (**A**) Cell viability test treated with LM and LM-FA particles using calcein-AM staining. (**B**) Confirmation of binding between cells and particles. (**C**) FACS, cell surface FOLR1 expression. (**D**) ELISA, P53 expression by cancer targeting. The difference in the expression increase in P53 was confirmed through ELISA. All statistical significance was confirmed for negative control. Non-significant values have been represented as ns, while *, **, ***, and **** indicate *p*-values < 0.0332, 0.0021, 0.0002, and 0.0001, respectively. In all figures, the HeLa cell is red; MDA-MB-231 is blue; and HDF is yellow.

To evaluate the effects of LM and LM-FA particles on cells, toxicity assays were performed. Based on the cytotoxicity of the LM particles confirmed above, the toxicity test of LM and LM-FA at 100 µg/mL concentration was conducted using calcein-AM. The viability was approximately 85% in the presence of LM and LM-FA particles compared to the negative control, confirming that cell viability was not affected by the particles (Figure 6A).

By utilizing the FOLR1 on the surface of cancer cells, we aimed to increase the efficiency of particle delivery and deliver the drug embedded in the particles. To accomplish this, we synthesized folate to be present on the surface of the particles and confirmed that it helps the particles bind to cancer cells. FACS was performed using an Alexa 488-conjugated FOLR1 antibody to determine FOLR1 expression on the cell surface and cell-line differences. We found that all cell lines expressed FOLR1. However, unlike MDA-MB-231 and HeLa, the expression of FOLR1 was significantly lower in HDF, a common cell line. Compared to MDA-MB-231 cells, the expression of FOLR1 is relatively high in HeLa cells (Figure 6B). This confirms the possibility that the folate receptor in cancer cells interacts with the folate on the surface of the particles to target cancer cells for drug delivery.

To determine whether the folate receptor on the surface of the cell and the folate on the surface of the particles affect binding, we found that LM and LM-FA particles bound to the cells better than did the negative control without particles. The particles with folate also bound better in the cancer cell lines MDA-MB-231 and HeLa. This is consistent with the expression trend of FOLR1 that we saw through FACS, and since the cancer cell lines expressed more folate receptors, we saw more binding when the particles had folate (Figure 6C).

Our particles utilize the folate on their surfaces to target folate receptors that are highly expressed on the surface of cancer cells. When bound to folate, the folate receptor transmits a signal into the cell, which in turn induces the expression of the intracellular protein P53 [30,31]. P53 is a classic tumor suppressor protein whose increased expression induces cells to enter the apoptotic phase, leading to increased cell death [32,33]. To determine the effect of LM-FA on the increased expression of P53, we treated cell lines with LM without folate and LM-FA particles with folate, and then checked the expression levels of P53 by ELISA. In HDF, a normal cell line, the expression of P53 was not increased, but in MDA-MB-231 and HeLa, more P53 was expressed in the LM-FA group than in the negative control (Figure 6D). This suggests that the particle binding to the folate receptor had an effect.

## 4. Conclusions

Folate receptors are also expressed on the surface of normal cells but are known to be overexpressed on the surface of cancer cells [34]. Different types of cancer cells express different amounts and types of proteins, and if a molecular marker can be identified, it will be easier to treat that cancer [35]. However, for cancers such as TNBC, which was the target of this study, no specific markers have been identified [36]. We developed LM-FA as a drug delivery vehicle and targeting particle applicable to the treatment of these diseases. The particles are approximately 300 nm in size, with a concentration of 1 × 10^9^ ± 4.9 × 10^6^ particles/mL, and are core–shell shaped with a phospholipid film on the surface to enhance stability. LM particles were evaluated for toxicity against HeLa, MDA-MB-231, and HDF cells and were found to be non-cytotoxic at concentrations below 250 µg/mL. In particular, LM-FA improved the efficiency of targeting cancer due to the presence of folate on the surface of cancer cells, thereby inducing an increase in the expression of P53, which helps to kill cancer cells.

Among the physical properties of EGaIn, we conducted experiments at the NIR wavelength of 855 nm, which is available to the human body, to confirm that the shape of the particles changes due to their photothermal properties [37,38]. We found that the presence of the particles increased the temperature by about 10 degrees and shortened the time it took for the heat to rise. The particles may be able to induce vascular embolization because they change shape when they reach a sufficient temperature [39,40]. Based on this characteristic, in future studies, we would like to see if we can block the blood vessels around the cancer to prevent blood supply to the cancer cells, thereby prolonging the drug to induce cancer cell death. Furthermore, since we have utilized folic acid to target cancer cells, we anticipate that it could also be used against other cancers, such as cervical cancer, skin cancer, and other types of breast cancer.

## Data Availability

Data available on request from the authors.

## Supplementary Materials

The following supporting information can be downloaded at: https://www.mdpi.com/article/10.3390/nano13132017/s1, Figure S1: LM particles characterization; Figure S2: SEM image and EDS mapping of LM-FA before and after NIR irradiation; Figure S3: Cell viability test of LM by CCK-8 assay; Figure S4: Cellular uptake of DOX by NIR irradiation.
