# Supplementary material for: Liquid-Metal Core–Shell Particles Coated with Folate and Phospholipids for Targeted Drug Delivery and Photothermal Treatment of Cancer Cells
Source: Nanomaterials (Basel). 2023 Jul 6;13(13):2017. doi: 10.3390/nano13132017 (PMC10343683; doi:10.3390/nano13132017)
Supplement: Supplementary file 1 [file nanomaterials-13-02017-s001.zip › nanomaterials-2411762-supplementary.docx]

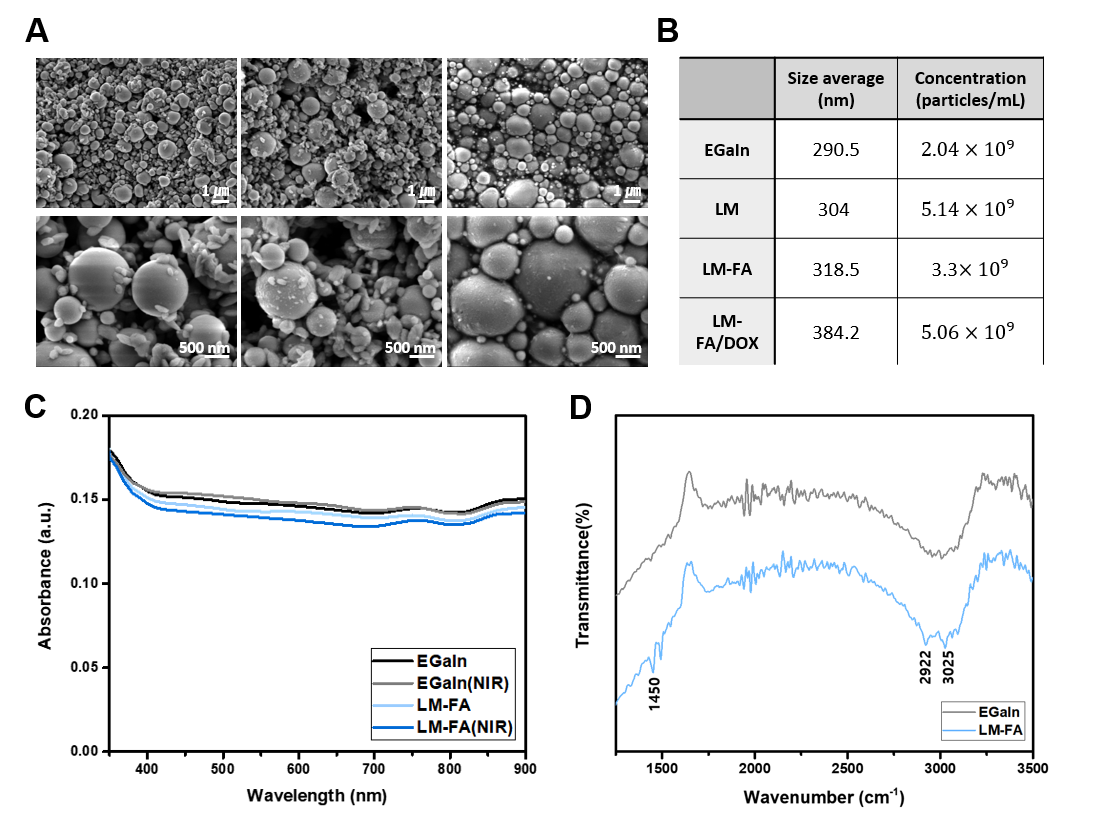


**Figure S1. LM particles characterization.** (**A**) SEM image of EGaIn, LM, LM-FA. (**B**) Concentration of particles from NTA. (**C**) UV-Vis-NIR absorbance spectrum of EGaIn and LM-FA before and after NIR irradiation. (**D**) FT-IR graph of EGaIn and LM-FA.


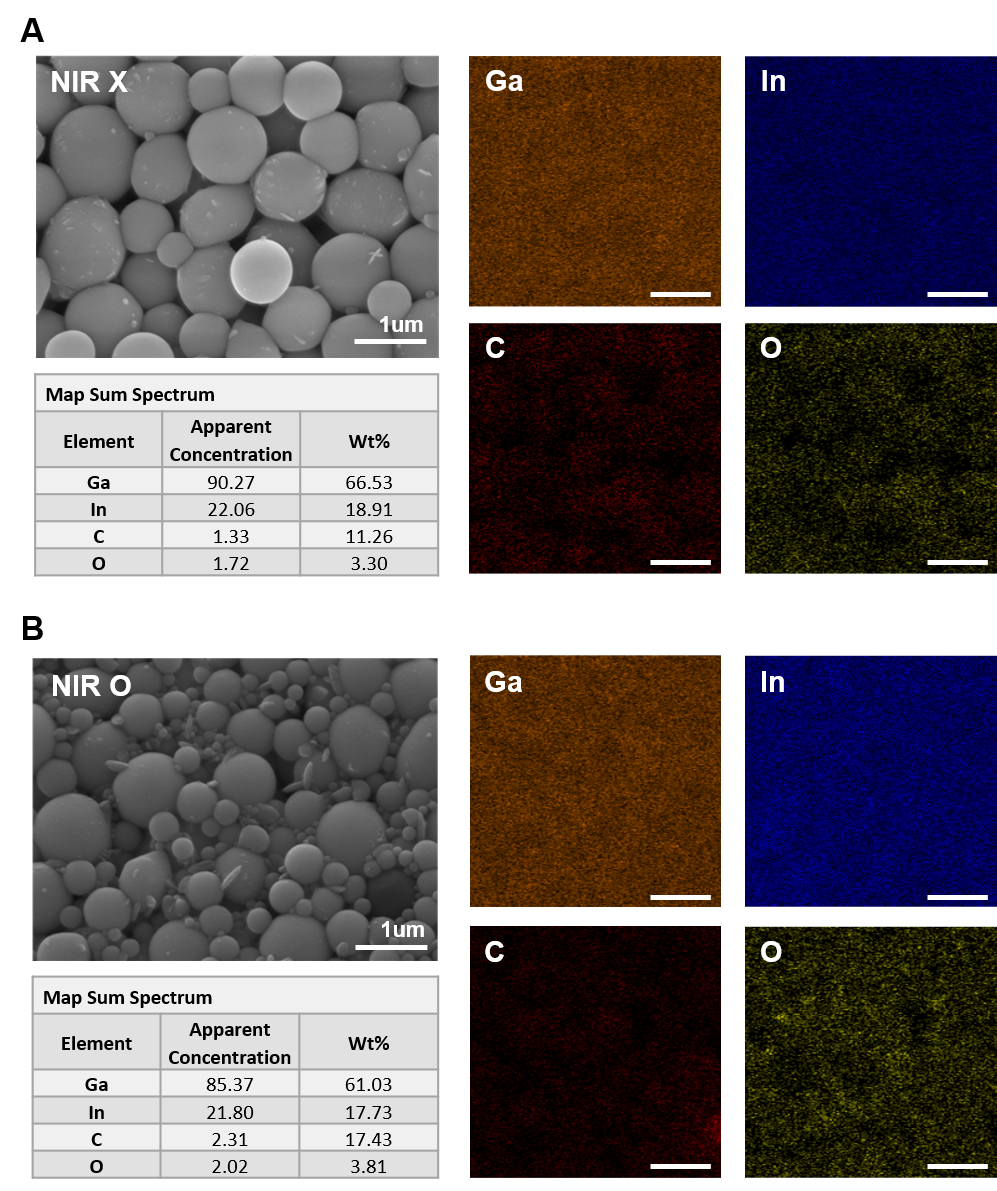


**Figure S2.** SEM image and EDS mapping of LM-FA before and after NIR irradiation. (**A**) LM-FA before NIR irradiation. (**B**) LM-FA after NIR irradiation.


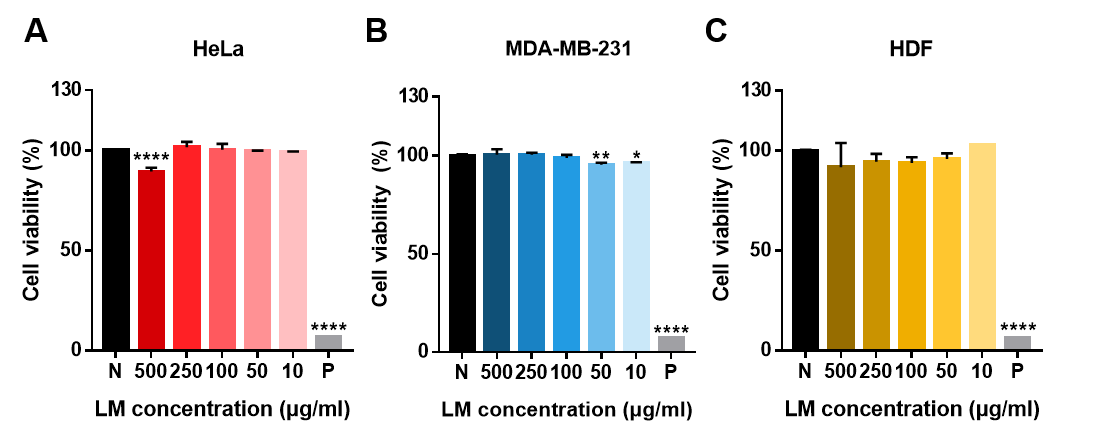


**Figure S3. Cell viability test of LM by CCK-8 assay.** (**A**) HeLa. (**B**) MDA-MB-231. (**C**) HDF. All statistical significance was confirmed for negative control. Non-significant values have been represented as ns, while *, **, ***, **** indicate p-values < 0.0332, 0.0021, 0.0002 and 0.0001, respectively.


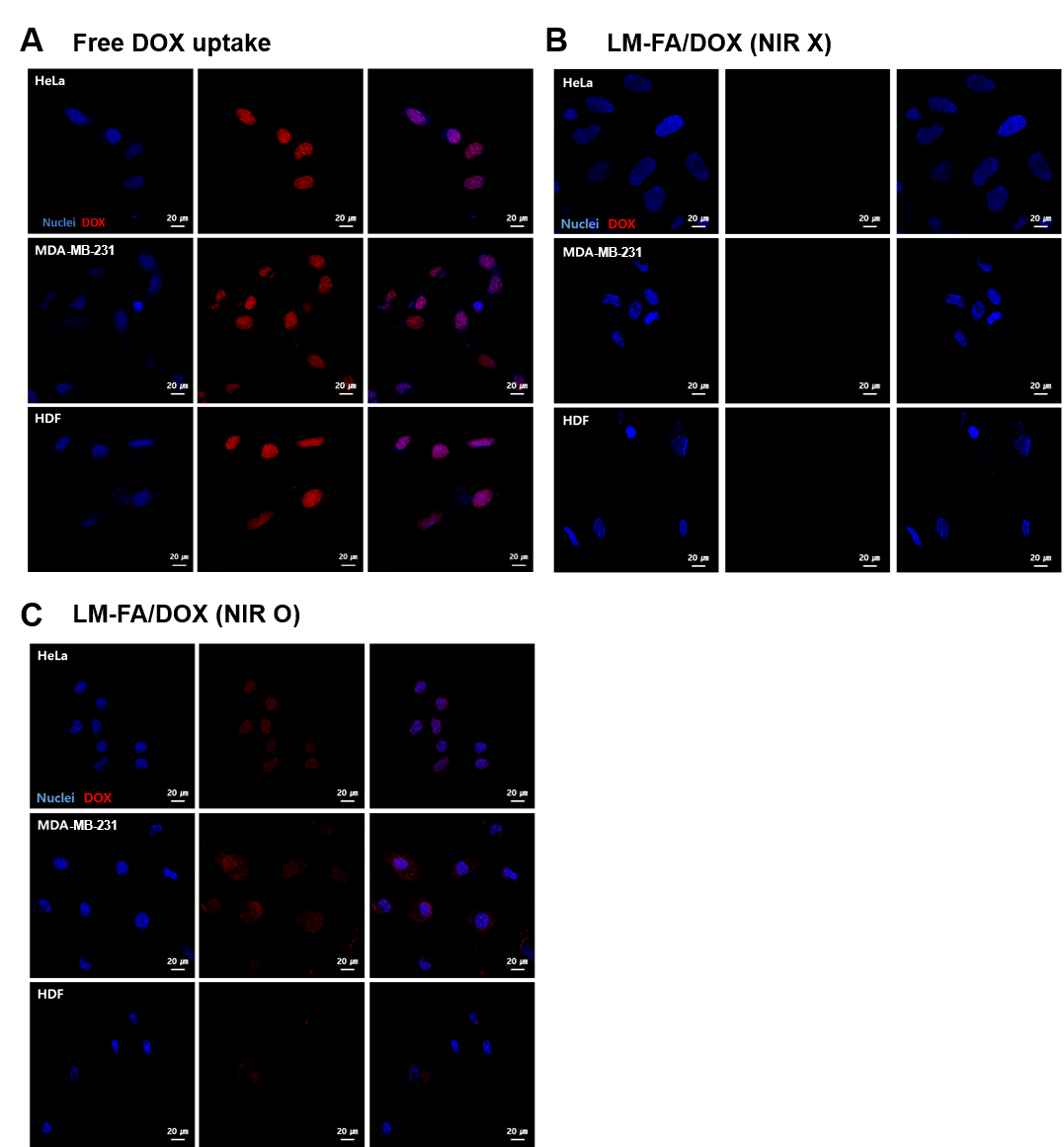


**Figure S4. Cellular uptake of DOX by NIR irradiation.** (**A**) Free DOX uptake. (**B**) DOX uptake by LM-FA/DOX particles. (No irradiation NIR) (**C**) DOX uptake released by NIR irradiation. From up HeLa, MDA-MB-231, and HDF cell.
